# *Asarum pubitessellatum*, sp. nov. (sect. *Heterotropa*, Aristolochiaceae) from Taiwan based on morphological and palynological evidence

**DOI:** 10.1186/1999-3110-54-28

**Published:** 2013-08-30

**Authors:** Chang-Tse Lu, Wen-Liang Chiou, Jenn-Che Wang

**Affiliations:** 1 Department of Life Science, National Taiwan Normal University, No. 88, Ting-Chow Rd., Sec 4, Wenshan, Taipei, 11677 Taiwan; 2 Botanical Garden Division, Taiwan Forestry Research Institute, No. 53, Nan-Hai Road, Chungcheng, Taipei, 10066 Taiwan

**Keywords:** *Asarum*, *Asarum crassisepalum*, *Asarum pubitessellatum*, *Asarum taipingshanianum*, *Heterotropa*, Pollen, Taiwan

## Abstract

**Background:**

Recently, we discovered an unknown *Asarum* from Taiwan which is closely related to *A. crassisepalum* S.F. Huang, T.H. Hsieh and T.C. Huang and *A. taipingshanianum* S.F. Huang, T.H. Hsieh and T.C. Huang by sharing a thick leaf blade, spreading perianth-lobes and a conical to cylindrical perianth-tube. We compared it with other related species and identified this plant as a new species.

**Results:**

This new species differs distinctly from the above two related species by having larger plant body, shortened rhizomes and an inner surface of the perianth-tube that is covered with numerous simple trichomes (vs. glandular trichomes). The pollen tectum in this new species is perforate, which differs from the incomplete reticulate with small supratectate granules in *A. crassisepalum* and the compact rugulate with small supratectate granules in *A. taipingshanianum*. Furthermore, these three species are geographically separated from one another.

**Conclusions:**

*Asarum pubitessellatum* C.T. Lu & J.C. Wang, a new species is described and illustrated. The trichomes on the inner surface of the perianth-tube and pollen micromorphology were the valuable characters in the low-level classification of *Heterotropa* species in Taiwan.

**Electronic supplementary material:**

The online version of this article (doi:10.1186/1999-3110-54-28) contains supplementary material, which is available to authorized users.

## Background

*Asarum* L. (Aristolochiaceae) consists of more than 100 species mainly distributed in the north temperate zone (Cheng and Yang 1983; Kelly 1998,2001; Huang et al.^2003^). Most species are distributed in eastern Asia, some species in North America, and one species is endemic to Europe. On the basis of the infrageneric classification proposed by Kelly (1998), this genus was separated into two distinct subgenera, each with two sections. The subgenus *Asarum*, composed of the sections *Asarum* and *Geotaenium*, is characterized by connate styles with terminal stigmas, inferior ovaries, and the inner surface of perianth-tubes puberulent to strigose; while the subgenus *Heterotropa*, comprising sections *Asiasarum* and *Heterotropa* (including *Hexastylis*), is characterized by six free styles with lateral stigmas, superior or half-inferior ovaries, and the inner surface of perianth-tubes longitudinal or with a strong network of ridges (Cheng and Yang 1983; Sugawara 1987; Kelly 1997).

Section *Heterotropa* is morphologically diversified and composed of ca. 70 species mainly distributed in eastern Asia, particularly in the Sino-Japanese region. However, because the perianth-tube is fleshy and brittle, distortion of the flower in pressed specimens makes its structure difficult to recognize. Consequently, herbarium specimens of *Heterotropa* are difficult to identify reliably leading to underestimates of the species diversity of the section. In Taiwan, the *Flora of Taiwan* 2^nd^ ed. recorded only four species in this section; however, our field expeditions in recent years have led to the discovery of 3 new species and 2 new records (Lu and Wang 2009; Lu et al. 2009; Lu et al. 2010).

Recently, Ms. Pi-Fong Lu discovered an unknown *Asarum* in Miaoli, Taiwan. We compared it with other related species and identified this plant as a new species based on the morphological and palynological evidence discussed here.

## Methods

The plants examined in this study were collected from native habitats and then transplanted into the greenhouse of National Taiwan Normal University, Taipei, Taiwan. The morphological and palynological data for *A. pubitessellatum* were based on the voucher specimens: TAIWAN. Miaoli Hsien: Mt. Chialishan, *C. T. Lu 812* (TNU) and same loc., *P. F. Lu 19112* (TNU). The palynological data of *A. crassisepalum* were based on the voucher specimens: TAIWAN. Hsinchu Hsien: Yuanyang Lake, *C. T. Lu 624* (TNU). The palynological data of *A. taipingshanianum* were based on the voucher specimens: TAIWAN. Ilan Hsien: Tsuifeng Lake, *C. T. Lu 743* (TNU).

Morphological Study—The measurement of floral characters was conducted using a Mitutoyo CD-6″CS digimatic caliper.

Pollen morphology—Pollen grains for scanning electron microscopic (SEM) study were collected from fresh anthers and prepared using the method proposed by Erdtman (1952). The acetolyzed grains were dehydrated through an ethanol series, critical point dried, coated with gold, and examined with a Hitachi SM 2400 scanning electron microscope. Descriptive terminology for pollen morphology follows that of Huang et al. (1995).

## Results

### Taxonomic treatment

**Figure 1:**
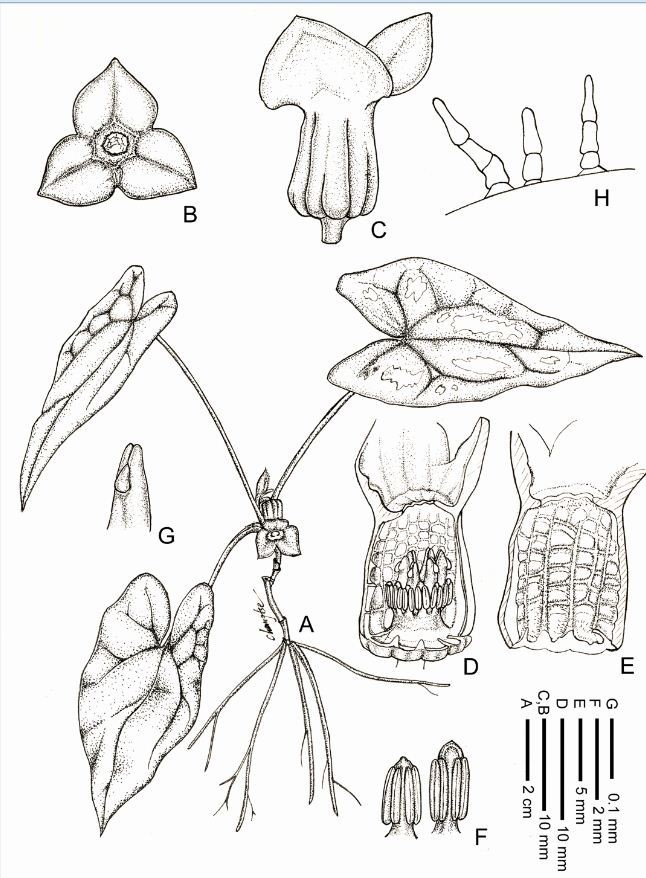
***Asarum pubitessellatum*** **. A**. Habit; **B**. Front view of flower; **C**. Perianth-tube; **D**. Dissected perianth tube, showing stamens and pistil; **E**. Part of perianth tube, showing tessellated inner surface; **F**. Stamens; **G**. Style and stigma; **H**. Trichomes on the inner surface of perianth tube. (from the holotype, *C. T. Lu, 812* (TNU)).

**Asarum pubitessellatum C.T. Lu** & **J.C. Wang**, sp. nov.—TYPE: TAIWAN. Miaoli Hsien: Nanchuang Township, Mt. Chialishan, alt. 1,400 m, 12 Dec. 2005, *C. T. Lu 812* (holotype: TNU; isotype: TAIF).  (Figure 1).

Diagnosis: *Asarum pubitessellatum* C.T. Lu & J.C. Wang is similar to *A. crassisepalum* S.F. Huang, T.H. Hsieh & T.C. Huang and *A. taipingshanianum* S.F. Huang, T.H. Hsieh & T.C. Huang but differs from the latter two by having larger plant body (20–30 cm vs. less than 10 cm tall); shortened rhizome; larger perianth-tube (ca. 1.5 cm vs. 1–1.2 cm in diam.); multicellular simple trichomes (vs. glandular trichomes) on the inner surface of perianth-tube.

Perennial herb. Rhizome short. Leaves 2 on each annual branchlet, with petiole 10–23 cm long. Leaf lamina triangular-ovate to sagittate, thick, 8.5–13 × 6–7.5 cm, acute to acuminate at apex, auriculate at base, base of the sinus 3–4.7 cm wide, glabrous adaxially, with white blotches along mid-vein, glabrous and pale green abaxially. Flowering branch with 2–3 cataphylls at its base, ovate, 16–18 mm long, margin ciliate, shed when leaves fully grown. Flowers solitary, facing downward, yellow-greenish to purple-greenish, perianth-tube conical, ca. 13–15 mm long, lower portion 12–13 mm in diam., upper portion 8–10 mm in diam., peduncle 10–18 mm long; outer surface glabrous, pale yellow-greenish and with numerous brownish red spots; internal surface purplish red, tessellated and with ca.12 longitudinal ribs, covered with numerous pubescences along longitudinal and transverse ribs. Tube throat slightly constricted, annual ca. 1–1.5 mm wide, orifice ca. 3–5 mm. Perianth-lobes 3, broadly triangular-ovate, yellow-greenish to purple-greenish, ca. 10–11 × 12–14 mm, spread, without forming semicircular pulvinate areas between the lobes and orifice. Stamens 12 in two whorls, filaments very short, anthers 2.5 mm long, with connective obtuse; ovary superior, 6-locular, styles 6, free, with slightly bifid apices; stigma oblong-ovoid, lateral, inserted in apex notch, extrorse; ovules 8 in each locule.

### Pollen morphology

**Figure 2:**
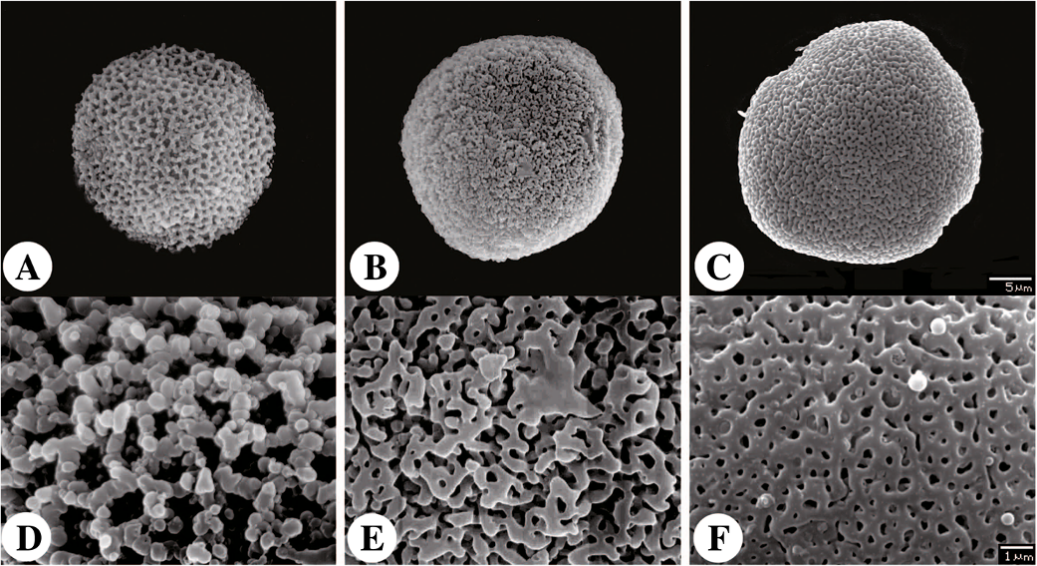
**Comparison of pollen morphology between** ***A. crassisepalum*** **(A, D),** ***A. taipingshanianum*** **(B, E) and** ***A. pubitessellatum*** **(C, F). A**-**C**. whole pollen. **D**-**F**. closed view of pollen tectum.

Pollen grains in *A. pubitessellatum* are oblate spheroidal to suboblate, penta-colporate in equatorial view (Figure 2C), ca. 25.5 × 27 μm–27 × 28.6 μm (P × E). Pollen tectum is perforate without supratectum granules (Figure 2F).

### Habitat and geographical distribution

**Figure 3:**
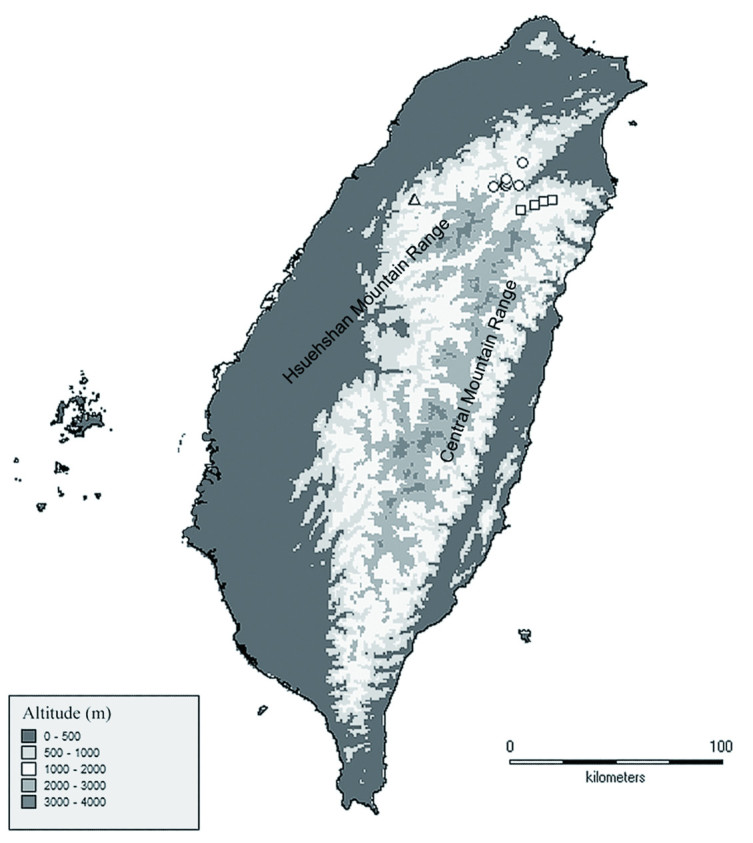
**Geographical distribution of** ***A. crassisepalum*** **(○),** ***A. taipingshanianum*** **(□), and** ***A. pubitessellatum*** **(△).**

To date, this new species is only known from the type locality in Mt. Chialishan, Miaoli Hsien, Taiwan. It was found under a shaded and moist plantation of the coniferous species *Cryptomeria japonica* (L.f.) D. Don. The habitat is similar to those of its allies, *A. crassisepalum* and *A. taipingshanianum*, which grow in mixed coniferous-broadleaf forests, often among mosses. These three species all occur in cloudy forest zones at middle elevation but in geographically different areas. *Asarum crassisepalum* inhabits the mountainous area around Yuanyang Lake in Hsuehshan Mountain Range, *A. taipingshanianum* inhabits the mountainous area of Taipingshan in the Central Mountain Range, and *A. pubitessellatum* can only be found on Mt. Chialishan on the western side of the Hsuehshan Mountain Range (Figure 3).

### Conservation assessment

Only two localities with less than 50 plant individuals of *A. pubitessellatum* have been found. The area of occurrence is estimated to be 10 km^2^. According to the IUCN red list categories (IUCN 2001) criteria, this species is categorized as critically endangered CR (B2abiii, C2a).

### Etymology

We name this species *A. pubitessellatum* based on the inner surface of its perianth-tube being covered with numerous simple trichomes along ridges. This character is different from other Taiwanese, Chinese, and Japanese *Heterotropa* species, which have perianth-tubes that are covered with glandular trichomes.

## Discussion

**Figure 4:**
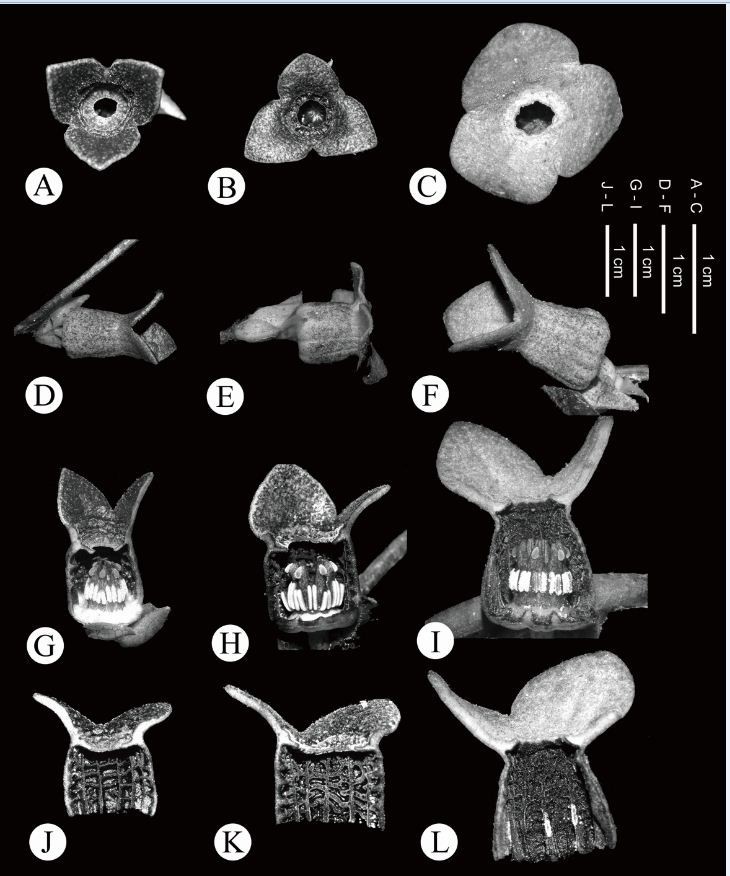
**Comparison of floral morphologies of** ***A. crassisepalum*** **(A, D, G, J),** ***A. taipingshanianum*** **(B, E, H, K) and** ***A. pubitessellatum*** **(C, F, I, L). A**-**C**. Front view; **D**-**F**. Perianth-tube; **G**-**I**. Dissected perianth-tube, showing anthers and pistils; **J**-**L**. Dissected perianth-tube, showing inner tessellated ridges.

**Figure 5:**
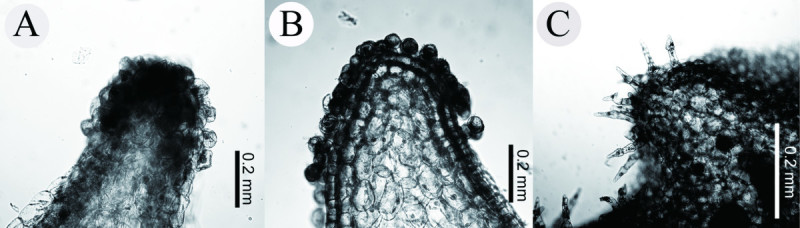
**Trichome types on the inner surface of perianth-tube. A**. *A. crassisepalum*; **B**. *A. taipingshanianum*; **C**. *A. pubitessellatum*.

Morphologically, *Asarum pubitessellatum* appears to resemble *A. crassisepalum* and *A. taipingshanianum*. These three species share the following common characters: lustrous, thick leaves, perianth-lobes spreading horizontally, perianth-tube only slightly constricted at the throat, inner surface with tessellated ridges, base of perianth-lobes smooth (lacking tubercles) or scarred by only a few lines (Figure 4). Despite these similarities, *A. pubitessellatum* is clearly distinguished from *A. crassisepalum* and *A. taipingshanianum* by the following characteristics: (1) larger plant body (20–30 cm high vs. usually less than 10 cm) with shorter rhizomes; (2) larger perianth-tube (ca. 1.5 cm vs. 1–1.2 cm) (Figure 4); and (3) multicellular simple trichomes on the inner surface of perianth-tube (vs. sessile unicellular glandular trichomes in *A. crassisepalum* and unicellular-stalked glandular trichomes in *A. taipingshanianum*) (Figure 5). A more detailed comparison between *A. pubitessellatum* and its allies, *A. crassisepalum* and *A. taipingshanianum* is given in Table 1 in aid of their identification. Species in sect. *Heterotropa* usually bear short-stalked, unicellular glandular trichomes on the inner surface of the perianth-tube (Sugawara 1987; Kelly 1997). However, it is normally not possible to preserve trichomes in herbarium specimens; therefore, this character has often been ignored by taxonomists. According to this study, we consider that the trichome type on the inner surface of perianth-tube can be a valuable character in the low-level classification of sect. *Heterotropa*.

**Table 1:** **Comparison of** ***Asarum pubitessellatum*** **and its allies,** ***A. crassisepalum*** **and** ***A. taipingshanianum***

	***A. crassisepalum***	***A. taipingshanianum***	***A. pubitessellatum***
**Rhizomes**	Elongated	Elongated	Short
**Leaves**			
Shape	Triangular-oblong to sagittate	Triangular-oblong	Triangular-ovate to sagittate
Size	4–9 × 3.5–6 cm	3–6 × 3.4–4.1 cm	8.5–13 × 6–7.5 cm
Apex	acute to acuminate	obtuse to acute	acute to acuminate
**Flower**			
Size (in diameter)	1–2 cm	1–2 cm	ca. 3 cm
Perianth-tube shape	Conical	Conical to cylindrical	Conical
Tubercle on the base of perianth-lobe	Scar-like	Absent or scar-like	Absent
Trichome type on the inner surface of perianth-tube	Sessile unicellular glandular trichome	Stalked unicellular-- glandular trichome	Multicellular single trichome
**Pollen micromorphology**	Incomplete reticulate with small supratectate granules	Compactly rugulate with small supratectate granules	Perforate

Checking the chromosome number of members of sect. *Heterotropa*, the species from Taiwan and Japan share the same basic chromosome number *x* = 12 (2*n* = 24, rare 36 or 48) rather than *x* = 13 (2*n* = 26 or 39, mainly distributed in southwestern China) (Huang et al. 1995; Kelly 1997; Lu and Wang 2009; Maekawa and Ono 1965; Ono 1960; Shi et al. 2008; Sugawara [Bibr CR24][Bibr CR25][Bibr CR26][Bibr CR28][Bibr CR29][Bibr CR30]; Sugawara and Ogisu 1992; Yinger 1983; Yuasa and Maekawa 1976), suggesting that the Taiwanese taxa are more closely related to the Japanese species than the Chinese species.

Pollen micromorphology is another valuable character in the low-level classification of the genus *Asarum* (Mi and Yang 1981; Huang et al. 1995; Lu and Wang 2009). Pollen exine ornamentation in sect. *Heterotropa* has been described as cerebelloid under verrucae for the Chinese species (Mi and Yang 1981), with rugulate-perforate, perforated subunits, incomplete reticulate or compact rugulate with large warts to small granules or none on supratectum for the Taiwanese species (Huang et al. 1995; Lu and Wang 2009; Lu et al. 2009; Lu et al. 2010) and microreticulate or microporate with gammae or verrucae or none for the North American species (as *Hexastylis*) (Niedenberger 2010).

The perforate pollen tectum without gammae or verrucae on the supertectum in *A. pubitessellatum* is quite different from its related allies: incomplete reticulate with small supratectate granules in *A. crassisepalum* (Figure 2A,D) and compact rugulate with small supratectate granules in *A. taipingshanianum* (Figure 2B,E). This type of pollen is similar to that of *A. hypogynum* and *A. chatienshanianum* from Taiwan (Huang et al. 1995; Lu and Wang 2009) and *A. naniflora* from North America (Niedenberger 2010). However, due to a lack of palynological information from the most diversified area, Japan, we cannot validate the significance of pollen morphology in the infra-section classification of sect. *Heterotropa*.

Geographically, *A. pubitessellatum*, *A. crassisepalum*, and *A. taipingshanianum* are currently distributed allopatrically, though they all occur in a similar habitat, under cypress forest [mainly composed of *Chamaecyparis formosensis* Matsum. or *C. obtusa* Sieb. & Zucc. var. *formosana* (Hayata) Rehder or both]. Their similar gross morphologies, pollen features, and habitat suggest that they are closely related. The contemporary geographic isolation of these three species may result from a decreased forest range or forest fragmentation. Further phylogeographical study is in progress to test this hypothesis.

The two editions of Flora of Taiwan (1975–1979; 1994–2003) and the Supplement to the Flora of Taiwan, 2nd ed. (Wang and Lu 2012) completely described Taiwan’s flora up to 2009. However, most recent findings, including new generic records, e.g., *Ypsilandra* (Hsu et al. 2011) and *Phacellanthus* (Chung et al. 2010), new species, e.g., *Cotoneaster rosiflorus* and *C. chingshuiensis* (Chang et al. 2011a, b), *Pouzolzia taiwaniana* (Peng et al. 2012), *Thismia huangii* (Chiang and Hsieh 2011), and *Tripterospermum hualiense* (Hsu and Chung 2012), and numerous newly recorded species, indicate that the documentation of the island’s vast and unique biodiversity is incomplete. This study echoes the suggestion of Hsu et al. (2011) and Peng et al. (2012) that the continuation of the botanical inventories is needed, especially those areas rarely botanized.

The following key is provided to distinguish the species of sect. *Heterotropa* in Taiwan.

Key to Taiwanese Species of *Asarum* sect. *Heterotropa* (modified from Lu and Wang 2009)

1. Leaves coriaceous, blades ovate, triangular-cordate to lanceolate-ovate; adaxial surface glabrous or sparsely hairy, dark green with white spots or maculate, abaxial surface glabrous or hairy along veins, light-green or purple; veinlet on abaxial surface indistinct. 2
2. Leaves subcoriaceous or chartaceous, blades triangular-ovate to broad ovate; adaxial surface sparsely hairy, dark green with white maculate, abaxial surface hairy along veins, green or purple; veinlets on abaxial surface distinct. 7
3. Plant erect; rhizome short; leaves longer than 7 cm; flowers 2–5 cm in diam. 3
4. Plant creeping; rhizome elongated; leaves less than 5 cm; flowers 1–2 cm in diam. 6
5. Leaves up to 30 cm; flowers ca. 3–5 cm in diam.; perianth-lobes longer than perianth-tube, throat constricted, neck-like; orifice rim developed, usually decurved, forming a funnel shape. *A. hypogynum*
6. Leaves less than 20 cm; flowers ca. 2–3 cm in diam.; perianth-lobes shorter than perianth-tube, throat constricted or slightly constricted, not neck-like; orifice rim present, not decurved. 4
7. Leaves lanceolate-ovate, margin undulate; throat constricted; perianth-tube length less than width. *A. tawushanianum*
8. Leaves triangular-ovate, margin entire; throat slightly constricted, perianth-tube length longer than width. 5
9. Leaf apex acute; outer surface of perianth-tube hairy, tubercles on base of perianth-lobes present, bar-like; filament attached to the base of perianth-tube. *A. yaeyamense*
10. Leaf apex acuminate; outer surface of perianth-tube glabrous, without tubercles on base of perianth-lobes; filament attached to the ovary. *A. pubitessellatum*
11. Perianth-tube conical or tubiform, length longer than width, lobes less than tube length; orifice less than 3 mm in diam. *A. crassisepalum*
12. Perianth-tube tubiform, length equal to width, lobes nearly equal to tube length; orifice more than 5 mm in diam. *A. taipingshanianum*
13. Perianth-tube pyriform; style laterally compressed, stigma unciform, terminal or subterminal; longitudinal ridges on inner surface 24 or more. 8
14. Perianth-tube tubiform or obconical; style not laterally compressed, stigma elliptic or lachrymiform, lateral; longitudinal ridges on inner surface 12–24. 9
15. Perianth-lobes longer than perianth-tube, with well developed tubercles on the base; orifice less than 3.5 mm in diam.; orifice rim well developed; inner surface of perianth-tube irregularly tessellated thoroughly, longitudinal ridges 24. *A. macranthum*
16. Perianth-lobes shorter than perianth-tube, with few tubercles on the base; orifice larger than 10 mm in diam.; orifice rim narrow; inner surface of perianth-tube irregularly tessellated on the upper half, but only longitudinal ribs on the lower half, longitudinal ridges more than 24. *A. satsumense*
17. Perianth-tube tubiform, length equal to width, inner surface of perianth-tube regularly tessellated, longitudinal ridges 12. *A. albomaculatum*
18. Perianth-tube obconical or tubiform, length longer than width, inner surface of perianth-tube irregularly tessellated, longitudinal ridges 12–24. 10
19. Perianth-tube tubiform, upper part slightly inflated; lobes yellow-greenish or purple-greenish, adaxial surface pubescent; outer surface yellow-greenish, inner surface purple. *A. chatienshanianum*
20. Perianth-tube obconical; lobes maroon, adaxial surface densely villous; flowers all maroon. *A. villisepalum*

## Conclusions

*Asarum pubitessellatum* C.T. Lu & J.C. Wang, a new species is described and illustrated based on the morphological and palynological evidence. The present study showed the trichomes on the inner surface of the perianth-tube and pollen micromorphology were the valuable characters to distinguish the closely related *Heterotropa* species in Taiwan.

## Authors’ original submitted files for images

Below are the links to the authors’ original submitted files for images. Authors’ original file for figure 1 Authors’ original file for figure 2 Authors’ original file for figure 3 Authors’ original file for figure 4 Authors’ original file for figure 5
