# Supplementary figures and images for: Asarum pubitessellatum, sp. nov. (sect. Heterotropa, Aristolochiaceae) from Taiwan based on morphological and palynological evidence
Source: Bot Stud. 2013 Aug 30;54:28. doi: 10.1186/1999-3110-54-28 (PMC5430370; doi:10.1186/1999-3110-54-28)

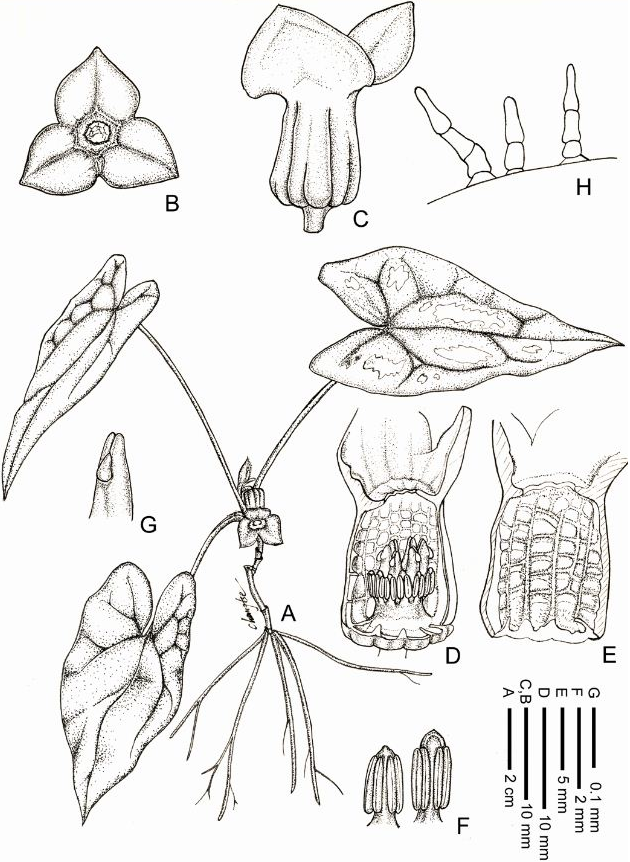

Supplement: Supplementary file 1 — Authors’ original file for figure 1 [file 40529_2012_23_MOESM1_ESM.tif]

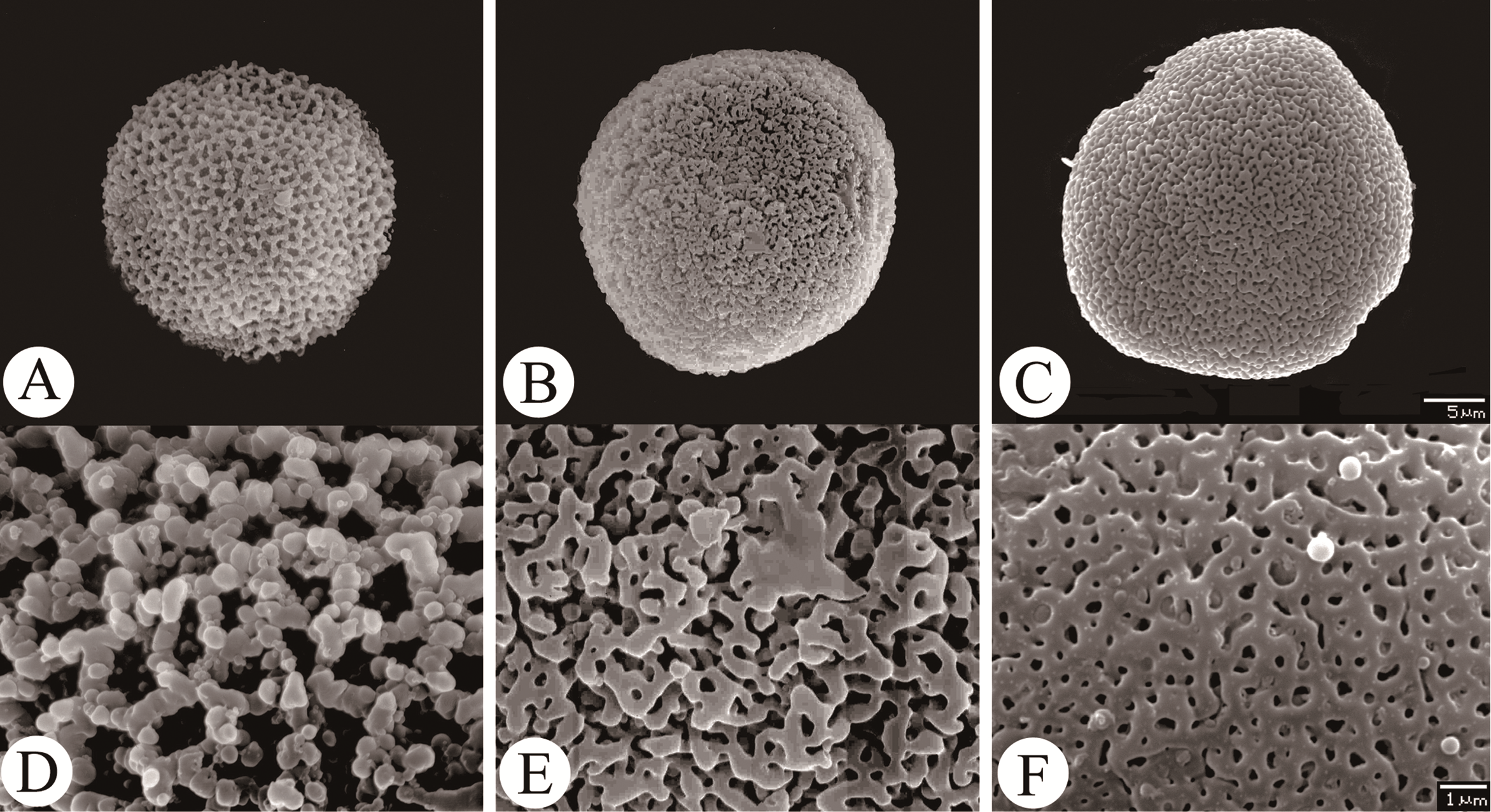

Supplement: Supplementary file 2 — Authors’ original file for figure 2 [file 40529_2012_23_MOESM2_ESM.tif]

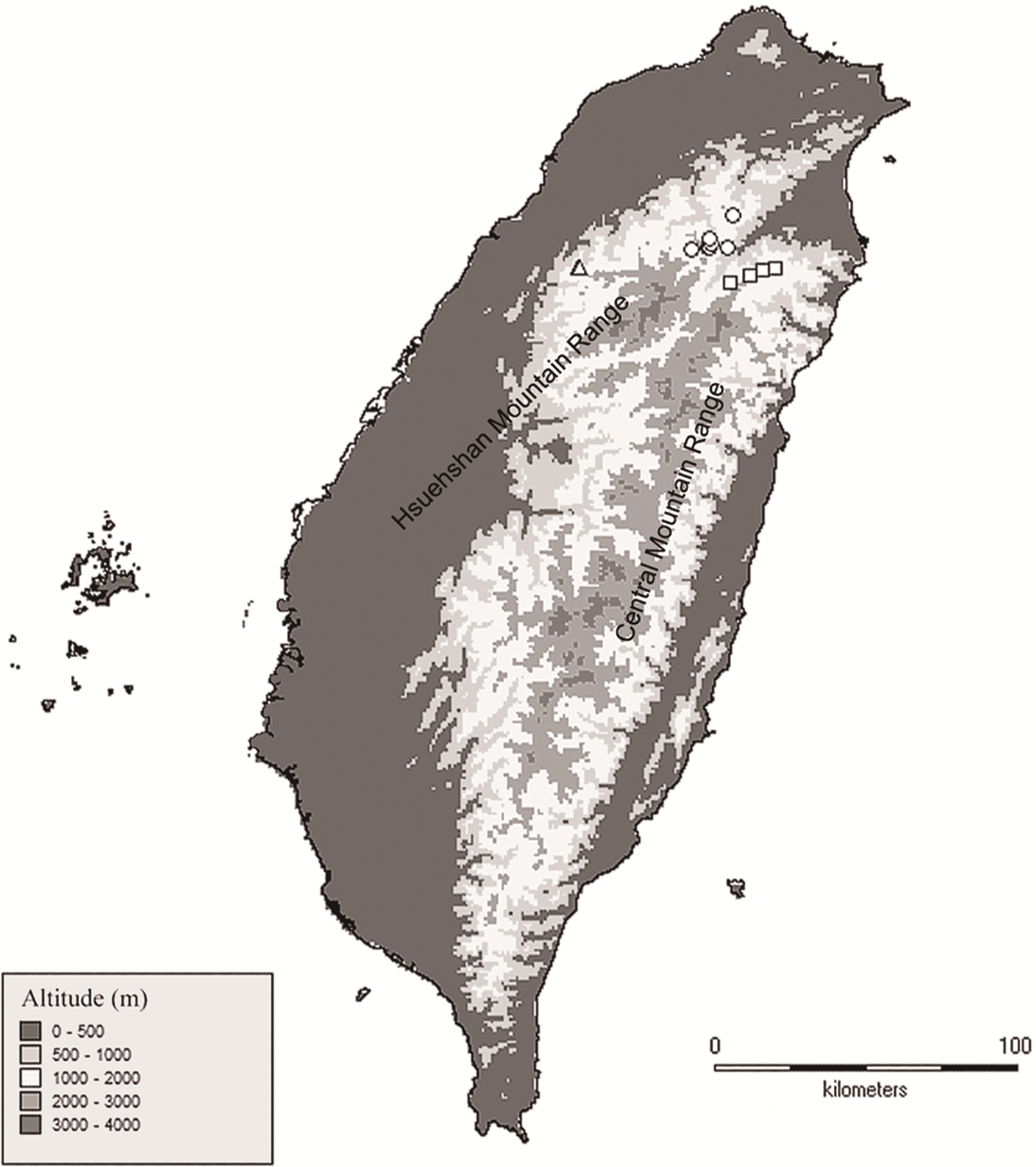

Supplement: Supplementary file 3 — Authors’ original file for figure 3 [file 40529_2012_23_MOESM3_ESM.tif]

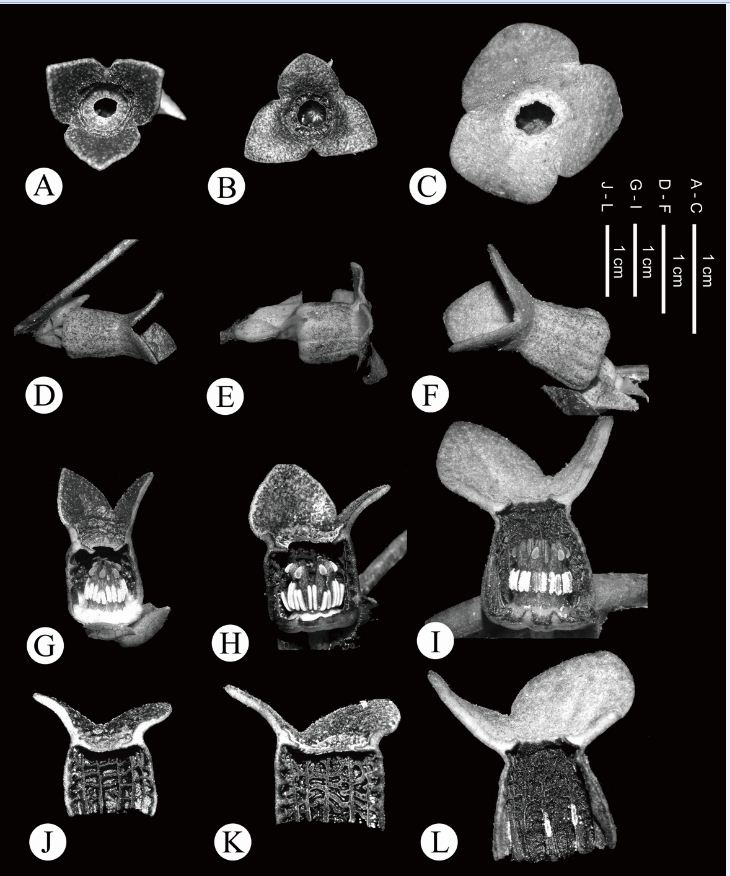

Supplement: Supplementary file 4 — Authors’ original file for figure 4 [file 40529_2012_23_MOESM4_ESM.tif]

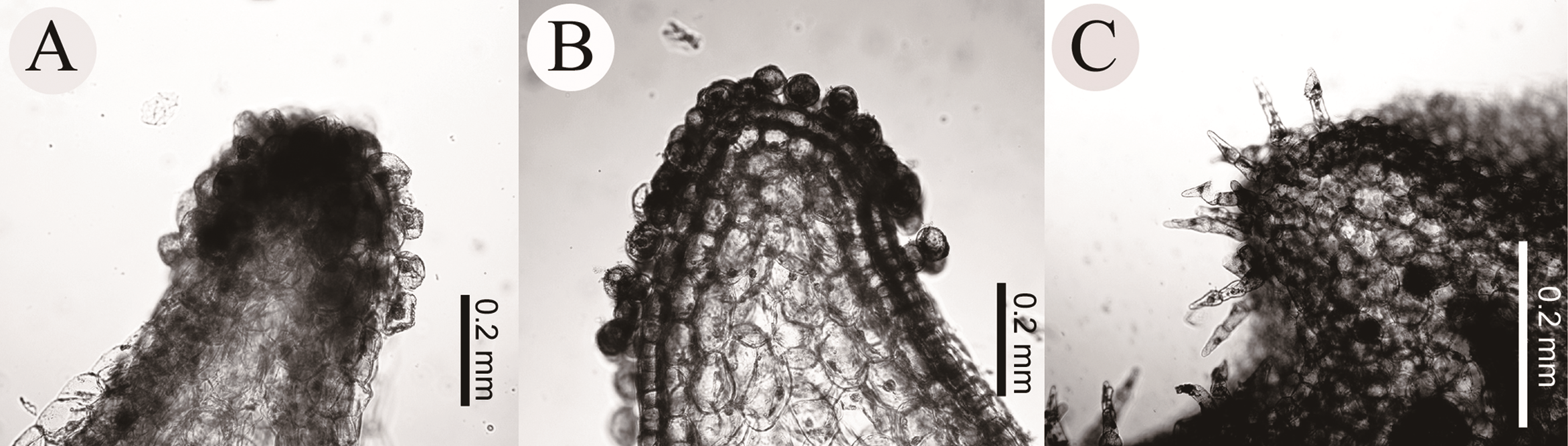

Supplement: Supplementary file 5 — Authors’ original file for figure 5 [file 40529_2012_23_MOESM5_ESM.tif]
